# Absence of carious lesions at margins of glass-ionomer cement and amalgam restorations: An update of systematic review evidence

**DOI:** 10.1186/1756-0500-4-58

**Published:** 2011-03-11

**Authors:** Steffen Mickenautsch, Veerasamy Yengopal

**Affiliations:** 1 Division of Public Oral Health, Faculty of Health Sciences, University of the Witwatersrand - 7 York Rd., Parktown/Johannesburg 2193, South Africa

## Abstract

**Background:**

This article aims to update the existing systematic review evidence elicited by Mickenautsch et al. up to 18 January 2008 (published in the European Journal of Paediatric Dentistry in 2009) and addressing the review question of whether, in the same dentition and same cavity class, glass-ionomer cement (GIC) restored cavities show less recurrent carious lesions on cavity margins than cavities restored with amalgam.

**Methods:**

The systematic literature search was extended beyond the original search date and a further hand-search and reference check was done. The quality of accepted trials was assessed, using updated quality criteria, and the risk of bias was investigated in more depth than previously reported. In addition, the focus of quantitative synthesis was shifted to single datasets extracted from the accepted trials.

**Results:**

The database search (up to 10 August 2010) identified 1 new trial, in addition to the 9 included in the original systematic review, and 11 further trials were included after a hand-search and reference check. Of these 21 trials, 11 were excluded and 10 were accepted for data extraction and quality assessment. Thirteen dichotomous datasets of primary outcomes and 4 datasets with secondary outcomes were extracted. Meta-analysis and cumulative meta-analysis were used in combining clinically homogenous datasets. The overall results of the computed datasets suggest that GIC has a higher caries-preventive effect than amalgam for restorations in permanent teeth. No difference was found for restorations in the primary dentition.

**Conclusion:**

This outcome is in agreement with the conclusions of the original systematic review. Although the findings of the trials identified in this update may be considered to be less affected by attrition- and publication bias, their risk of selection- and detection/performance bias is high. Thus, verification of the currently available results requires further high-quality randomised control trials.

## Introduction

Carious lesions associated with the margins of tooth restorations have been defined as recurrent or secondary caries [1]. In recent years it has been suggested that placing a filling does not cure caries and that the "recurrence" of lesions on restoration margins results from neglecting to treat caries as a disease before placing a restoration [2]. Part of the treatment of caries is encouraging remineralisation in the cavity walls [3]. Ten Cate and van Duinen [4] have shown, in-situ, a hyper-remineralisation effect in demineralised tooth tissues bordering glass ionomer cement (GIC) type restorations. The significant remineralisation potential of GIC has been ascribed to the release of fluoride ions, facilitated by a hydrophilic environment [5]. In addition, the release of strontium by GIC and its diffusion into demineralised tooth tissue, thus further aiding remineralisation, has been observed [6]. Moreover, it has been suggested that carious lesions are rarely the cause of GIC restoration failures [1].

Mickenautsch et al. [7], in a previous systematic review with meta-analysis, first reported on the combined results of trials comparing the absence of carious lesions at margins of GIC and amalgam restorations. This meta-analysis was limited, owing to a low number of identified randomised control trials. It concluded that after 6 years the absence of carious lesions at margins of single-surface GIC restorations was higher than their absence on amalgam fillings of permanent teeth. Results for both multiple- and single-surface restorations in primary teeth showed no difference between the two materials.

The results of this meta-analysis were based on a systematic search of literature until 5 January 2008 [7]. It has been suggested that once the search date of a systematic review is older than even 1 year, users should check for more recent trials on the same topic to see whether new evidence has altered the findings of a given systematic review [8]. In addition, the original quality assessment criteria [7] may be questioned on grounds of being ineffective in judging the true internal validity of trials on basis of risk of bias [9,10]. Therefore, the aim of this update is to provide a more in-depth assessment of bias-risk in trials.

Thus, this quantitative systematic review aims to update the existing evidence provided by the original article [7] regarding the review question of whether, in the same dentition and same cavity class, glass-ionomer cement (GIC) restored cavities show less recurrent carious lesions on cavity margins than cavities restored with amalgam.

## Materials and methods

In order to update the existing evidence, the systematic literature search was extended from the original search date and a further hand-search and reference check were done. Updated quality criteria were used in assessing the quality of accepted trials (Table 1) [9-12] and the risk of bias was investigated in more depth than in the previous report. In addition, the focus of quantitative synthesis was shifted to single datasets (DS) extracted from the accepted trials.

**Table 1:** Quality assessment criteria of trials

Selection bias		
**Score**	**Criteria**	**Impact on bias risk**
Randomisation and concealment		
A	(i) Randomisation: Details of any adequate type of allocation method that generates random sequences with the patient as unit of randomisation are reported.^1^ (ii) Concealment: Trial provides evidence^2 ^that concealment was indeed effective and that the random sequence could not have been observed or predicted throughout the duration of the trial.	Doubts may still exist whether the trial results are influenced by selection bias but no indication can be found from the trial report to support such doubt.
B	(i) Randomisation: Details of any adequate type of allocation method that generates random sequences with the patient as unit of randomisation are reported.^1^ (ii) Concealment: Trial reports on any adequate method to prevent direct observation^3 ^and prediction^4 ^of the allocation sequence and sequence generation rules.	Despite the implementation of method considered to be able to prevent unmasking of the concealed allocation sequence through direct observation and prediction, there are reasons to expect that the concealed allocation sequence may have been unmasked during the cause of the trial.
C	(i) Randomisation: Details of any adequate type of allocation method that generates random sequences with the patient as unit of randomisation are reported.^1^ (ii) Concealment: Trial reports on any adequate method to prevent direct operator observation of allocation sequence and sequence generation rules^3^. However, the allocation sequence and sequence generation may have been sufficiently predicted.	Despite the implementation of method considered to be able to prevent unmasking of the concealed allocation sequence through direct observation, there are reasons to expect that operators could have predicted the concealed allocation sequence.
D	(i) Randomisation: Details of any adequate type of allocation method that generates random sequences with the patient as unit of randomisation are reported.^1^ (ii) Concealment: The trial report does not include information on how the allocation of random sequence was concealed. The allocation could have been directly observed and/or predicted.	Despite the theoretical chance for each patient to be allocated to either treatment group, operator knowledge of the allocation sequence may have lead to patient allocation that favoured the outcome of one type of treatment above the other.
0	Trial does not comply with criteria A - D.	No guaranty of equal chance for patients to be allocated to either treatment group, thus allocation may have favoured the outcome of one type of treatment above the other.
Baseline data for randomised trials		
A	Baseline data collected before randomisation and reported for both treatment groups. Data shows no significant differences between both groups.	Evidence is given that randomisation has lead to equal groups suggesting little risk of selection bias.
B	Baseline data collected before randomisation and reported for both treatment groups. Data shows significant differences between both groups but has been statistically adjusted appropriately.	Differences have been adjusted, thus the influence of possible selection bias appears to be reduced.
C	Baseline data collected before randomisation and reported for both treatment groups. Data shows significant differences between both groups without being statistically adjusted.	Reported differences may be due to ineffective randomisation, thus indicate risk of selection bias.
0	Trial does not comply with criteria A - C.	No evidence is given whether randomisation has indeed lead to equal groups with differences beyond chance, thus differences may exists indicating selection bias.
Detection/Performance bias		
Blinding/Masking		
Score	Criteria	Impact on bias risk
A	(i) Trial reports on any type of method that is known to prevent patient AND operator AND evaluator to discern whether patients are allocated to the test- or the control group (Blinding/Masking). (ii) Trial reports a process with which the effect of Blinding/Masking was evaluated, as well as the results of such evaluation.	Evidence is given that the trial results may not have been influenced by detection/performance bias that may have favored the outcome of one type of treatment above the other.
B	(i) Trial reports on any type of method that is known to prevent patient AND operator AND evaluator to discern whether patients are allocated to the test- or the control group (Blinding/Masking). (ii) Trial report does not give reason for doubt that the patient allocation to either the test- or the control group has been unmasked throughout the duration of the trial.	Doubts may still exist whether the trial results are influenced by detection/performance bias but no indication can be found from the trial report to support such doubt. However, no evaluation of the Blinding/Masking effect has been included in the trial, thus no evidence for lack of bias is given.
C	(i) Trial reports on any type of method that is known to prevent patient AND operator AND evaluator to discern whether patients are allocated to the test- or the control group (Blinding/Masking). (ii) Trial report gives reason for doubt that the patient allocation to either the test- or the control group has been unmasked throughout the duration of the trial.	Despite the implementation of method considered to be able to prevent unmasking, there are reasons to expect that operators/patients could have discovered the allocation.
0	No process reported or implemented able to blind/mask patients AND operators whether patients where allocated to either the test- or the control group (It is insufficient to report that blinding/masking was done without reporting the details of the process).	Knowledge about the patient allocation may have caused patients/operator to act in a way that may have favoured the outcome of one type of treatment above the other,
Attrition bias		
Loss - to follow up		
Score	Criteria	Impact on bias risk
A	Available case analysis, loss-to-follow up reported per treatment group. Subsequent sensitivity analysis does not indicate a possible risk of bias.	The trial allows extracting evidence that attrition may not have favoured the outcome of one type of treatment above the other.
B	Available case analysis, loss-to-follow up reported per treatment group. Subsequent sensitivity analysis indicates a possible risk of bias.	The trial allows assessing the risk that attrition may have favoured the outcome of one type of treatment above the other.
0	Trial does not report number of included participants per treatment group at baseline or gives any indication that would allow ascertaining the loss-to-follow up rate per treatment group.	The trial carries an unknown risk that attrition may have favoured the outcome of one type of treatment above the other.
Run-in phase		
A	No run-in phase reported or discernable during which patients were given the active treatment or the placebo/control.	The trial may not carry the risk of bias due to exclusion of patients who would not respond well to e.g. the active treatment.
0	Run-in phase reported or discernable during which patients were given the active treatment or the placebo/control.	During a run-in phase only patients were selected for randomisation that have responded/not responded to the active treatment of the placebo/control. This may favour the outcome of one type of treatment above the other as patients who did not respond well to either are excluded.
Trial endpoints		
0	The trial reports on secondary or surrogate outcomes as endpoints.	Even if the surrogate results would highly correlate with primary (i.e. clinical) outcomes, they cannot serve as valid replacements and need to be regarded for hypothesis development, only.
A	The trial reports on primary outcomes as endpoints.	Primary outcomes may provide evidence for hypothesis testing.

^1 ^Excluded are types of allocation methods that are considered as inadequate: cluster randomisation, fixed block randomisation with block size 2, minimisation, alternation, randomisation of teeth, use of date of birth or patient record number, "quasi"-randomisation, split-mouth

^2 ^E.g. by reporting results of the Berger-Exner Test or any other statistical tests that show that covariates of compared groups were similar at baseline

^3 ^E.g. by opening of opaque envelope, obtaining allocation from tables, computer generated or from other sources

^4 ^E.g. central randomisation, sequence allocation by other than operator; excluding varied block randomisation

### Literature search, review and quality assessment of trials

The search strategy used in the previous review [7] was replicated for this review update, using the search terms: *Dental Caries OR Dental Caries Susceptibility OR Root Caries OR Tooth Demineralization AND Glass Ionomer Cements OR Cermet Cements AND Cariostatic Agents OR Dental Caries OR Cariostatic Agents AND Dental Amalgam OR silver mercury amalgam *and *Ionomer$ and amalg$ and cariosta$*. Only the start and cut-off dates were changed. The databases Biomed Central, Cochrane Oral Health Reviews, Cochrane Library, Directory Of Open Access Journals, Expanded Academic ASAP PLUS, Meta Register Of Controlled Trials, PubMed and Science-Direct, were searched for relevant papers published between 8 January 2008 (the search cut-off date of the original systematic review) and 10 August 2010. Criteria for trial inclusion were:

- 2-arm clinical prospective study design;

- Comparison of GIC versus Amalgam;

- Year of publication of trials, identified through hand-search/reference check, from 1990.

Included trials were excluded after further review if:

- No outcome measure related to caries was reported;

- No computable data, consisting of number of teeth included at baseline (BSL), number of observed effects (n) and total number of evaluations (N), per treatment group was reported.

Included trials that passed the exclusion criteria were accepted for further quality assessment and data extraction. Review, data extraction and quality assessment of the accepted trials was undertaken independently by two reviewers (SM and VY). Differences were resolved through discussion and consensus.

In contrast to the original published systematic review [7], quality assessment of accepted trials was undertaken on the basis of availability of evidence indicating successful prevention of selection- and detection/performance bias from start to end of each trial. The new criteria (Table 1) differ from those used in the first review [7]. It has been argued that the inclusion of bias-preventing measures (e.g. randomisation, blinding/masking) into the trial methodology only demonstrates an attempt to reduce bias risk but does not carry proof in itself that such attempt was indeed successful and that it is far more important to judge trial quality according to evidence that indicates to what extent such attempt has succeeded [9]. Against this background the quality criteria were adjusted accordingly. Thus, where trials only reported that randomisation was conducted or included a detailed description of the randomisation process, this was not considered adequate if they failed to provide any evidence that randomisation was indeed effective throughout the trial.

Potential attrition- and publication bias was not investigated in the original systematic review [7]. This update used RevMan Version 4.2 statistical software by The Nordic Cochrane Centre, The Cochrane Collaboration (Copenhagen; 2003) in conducting sensitivity analysis in order to investigate potential risk of attrition bias in trials. To investigate publication bias, a funnel plot was generated, using the datasets from the included clinical trials. The standard error (SE) of the Mean differences was plotted on the Y-axis, and the log of the Relative Risk (RR) on the X-axis, using MIX Version 1.7 meta-analysis software [13]. In addition, the Egger's linear regression method [14] was used to calculate an intercept with 95% Confidence Interval (CI) with statistical significance set at α = 0.05.

### Data extraction and analysis

All data concerning primary and secondary outcomes of accepted trials were extracted as single dichotomous datasets, containing the number of observed effects (n) and the total number of evaluations (N) for both the control and the test groups. The Cochrane RevMan, Version 4.2 software package was used in computing the Relative Risk (RR, 95% CI). Statistical significance was set at α = 0.05.

Meta-analysis, using RevMan Version 4.2 statistical software by The Nordic Cochrane Centre, The Cochrane Collaboration (Copenhagen; 2003), was considered for datasets only if they complied with the previously published criteria for clinical homogeneity [7]. The percentage of total variations across datasets (I^2^) was used in assessing statistical heterogeneity [15]. Statistical significance for assessing statistical heterogeneity was set at α = 0.10. A fixed-effects model was used for meta-analysis under condition of statistical homogeneity of datasets. Pooled datasets were assigned Mantel-Haenszel weights directly proportionate to their sample sizes.

Cumulative meta-analysis, using MIX Version 1.7 meta-analysis software [13], was performed for datasets of consecutive follow-up periods, which also showed clinical and statistical homogeneity, in order to investigate whether a chronological trend of the available evidence might be observed.

## Results

### Literature search

**Figure 1:**
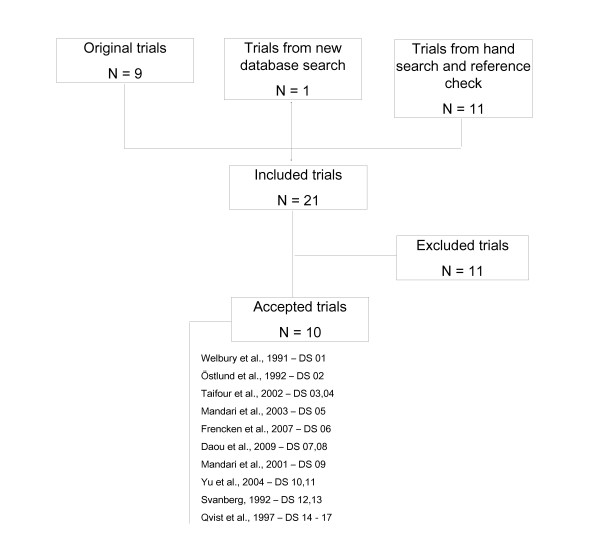
**Flow diagram of trial selection**. N = Number of trials; DS = Dataset number.

In addition to the 9 trials included in the original meta-analysis [16-24], one trial [25] was identified during the new database search and a further 11 [26-36] from the hand-search and reference check were included (Figure 1). Of these 21 trials, 11 were excluded [16-19,30-36], for reasons shown in Table 2. Ten trials passed the exclusion criteria and were accepted for data extraction and quality assessment [20-29]. Of the accepted trials two were of parallel group design [22,24] and five were split-mouth studies [20,23,27-29]. Three trials used the tooth and not the patient as unit of randomisation [21,25,26] and can be regarded as of partial split-mouth design: in one trial 20 out of 50 patients received more than one type of restoration [21]; during one trial 45 patients were treated with 38 amalgam and 35 GIC restorations [25] and in one further trial 92 out of 666 patients received both types of materials [26].

**Table 2:** Excluded trials with reasons for exclusion

Article	Reason for exclusion
Hickel and Voss, 1990 [31]	Does not report on caries as trial outcome
Frencken et al., 2006 [33]	Does not report on caries as trial outcome
Smith et al., 1990 [34]	Does not report on caries as trial outcome
Smales et al., 1990 [35]	Does not report on caries as trial outcome
Yip et al., 2002 [36]	Does not report on caries as trial outcome
Phantumvanit et al., 1996 [16]	No computable data reported: Does not report on number of same type of units for caries (tooth surfaces) as total number of evaluated units (restored teeth), baseline number of units
Rahimtoola and van Amerongen, 2002 [17]	No computable data reported: Does not report on number of evaluated GIC/Amalgam restorations
Taifour et al., 2003 [18]	No computable data reported: Does not report on number of baseline, restorations, number of evaluated restorations, number of restorations with caries (GIC/Amalgam)
Qvist et al., 2004 [19]	No computable data reported: Does not report on number loss-to-follow up restorations per GIC/Amalgam
Rahimtoola and van Amerongen, 1997 [30]	Published study protocol-no results reported
Mjör and Jokstad, 1993 [32]	No computable data reported: Number of carious teeth not reported as quantitative units per GIC/Amalgam

GIC = Glass-ionomer cement.

### Data extraction and analysis results

Thirteen individual computable datasets (DS 01-12,14) of primary outcomes and 4 datasets with secondary outcomes (DS 13,15-17) were extracted from the 10 accepted trials. Characteristics of these trials and their datasets are shown in Table 3. It has to be noted that the two trials by Mandari et al., 2001 and 2003 [23,27], as well by Taifour et al., 2002 and Frencken et al., 2007 [22,24] report each of different datasets from the same trials.

**Table 3:** Details of accepted trials

Article	DS	Patient character-istics/potential confounders*	GIC treatment group					Amalgam treatment group					Outcome measure	Evaluation		Dentition/Teeth/Restoration	Study period
							
			Type of material	BSL	N	n	LTF	Type of material	BSL	N	n	LTF		Criteria	Method		
Welbury et al., 1991 [20]	01	^[1]^	Ketac Fil	119	51	7	68	Amalcap	119	51	11	68	Recurrent caries	USPHS	Clinical examination	Primary/Molars/Class I & II	5 years
Östlund et al., 1992 [21]	02	^[2]^	Chem Fil	25	10	0	15	ANA 2000	25	23	1	2	Recurrent caries	USPHS	Clinical examination	Primary/Molars/Class II	3 years
Taifour et al., 2002 [22]	03	^[3]^	Fuji IX/ Ketac Molar	610	475	9	135	Avalloy	425	331	11	94	Caries on margin	ART	Clinical examination	Primary/Molars/Single surface	3 years
	04			478	106	4	372		380	84	9	296				Primary/Molars/Multiple surface	
Mandari et al., 2003 [23]	05	^[4]^	Fuji II	223	173	3	50	ANA 2000	207	162	16	45	Recurrent caries	Modified USPHS	Clinical examination	Permanent/Molars/Single surface	6 years
Frencken et al., 2007 [24]	06	^[5]^	Fuji IX/ Ketac Molar	487	153	11	334	Avalloy	403	108	15	295	Caries on margin	ART	Clinical examination	Permanent/Molars/Single surface	6.3 years
Daou et al., 2009 [25]	07	^[6]^	Fuji IX	35	33	4	2	Permite C	38	36	1	2	Recurrent caries	USPHS	Clinical examination	Primary/Molars/Class I & II	1 year
	08			35	23	3	12		38	21	3	17					2 years
Mandari et al., 2001 [27]	09	^[7]^	Fuji II	223	211	9	12	ANA 2000	207	196	15	11	Recurrent caries	Modified USPHS	Clinical examination	Permanent/Molars/Single surface	2 years
Yu et al., 2004 [28]	10	^[8]^	Fuji IX/ Ketac Molar-aplicap	45	37	0	8	GK amalgam	32	23	0	9	Recurrent caries	ART	Clinical examination	Primary/Molars/Single surface	1 year
	11			45	29	0	16		32	18	0	14					2 years
Svanberg, 1992 [29]	12	^[9]^	Ketac Silver	18	14	0	4	Disper-salloy	18	14	3	4	Recurrent caries	SNBHW	Clinical examination	Permanent/Molars & Premolars/GIC = Tunnel/Amalgam = Class II	3 years
	13			11	11	3	0		11	11	9	0	Caries progression		Probing & Bitewing	Permanent/approximal adjacent surfaces	
Qvist et al., 1997 [26]	14	^[10]^	Ketac Fil	515	334	11	181	Disper-salloy	543	306	17	237	Recurrent caries	DPDHS	Clinical examination	Primary/Class I, II and III/V	3 years
	15			127	105	25	22		127	94	47	33	Caries progression			Primary & permanent/approximal adjacent surfaces (sound or arrested caries)	
	16			156	120	25	36		183	129	47	54	Caries progression			Primary/approximal adjacent surfaces (carious or active lesion)	
	17			156	120	66	36		183	129	78	54	No caries regression			Primary/approximal adjacent surfaces (carious or active lesion)	

DS = Dataset number; BSL = Number of teeth at baseline; N = Number of teeth evaluated; n = Number of teeth with caries, LTF = Loss-to-follow-up; USPHS = United States Public Health Service criteria; ART = Criteria for atraumatic restorative treatment; SNBHW = Criteria according to the Swedish National Board for Health and Welfare; DPHS = Danish Public Dental Health Service criteria.

* Potential confounders = Reported fluoride exposure; high-sugary diet; poor oral hygiene; high past caries experience.

Patient characteristics:

[1] Split-mouth trial. 76 patients, age 5 - 11 years; patients attending the Department of Child Dental Health at Newcastle Dental Hospital (UK) for routine restorative care; subjects were admitted to the trials if they required at least 1 pair of restorations in their deciduous molar dentition; paired cavities either Class I or II, if possible in the same tooth type; restoration always in different quadrants per pair; any cavity was suitable for inclusion; a cavity was excluded if it could only be satisfactory restored using a stainless steel crown; restorations placed between October 1982 and March 1987;caries removal by drill.

**Potential confounders reported: **none.

[2] Partial split-mouth trial. 56 patients, age 4-6 years regularly treated at one Public Dental Service clinic in Jönköping, Sweden who showed manifest caries lesion on the mesial surface of a 2^nd ^primary molar; lesion not atypical or extended into buccal or lingual tooth surfaces; lesion completely surrounded by healthy enamel and should not reach the pulp; caries removal by drill;

**Potential confounders reported: **none.

[3] Parallel group trial. 835 patients, age 6-7 years from Damascus, Syria; with dentinal lesions with an opening wide enough for the smallest excavator to enter (diameter = 0.9 mm, without pulp involvement; size of restorations varied from small to large; dental caries prevalence 85%; mean dmfts and dmft scores of molars plus canines 9.0 and 4.4, respectively; GIC restorations placed after caries removal by hand excavation (ART).

**Potential confounders reported: **High past caries experience.

[4] Split-mouth trial. 152 patients from a cohort of grade 3-5 pupils, mean age 11 years in need of 2 or more restorations; from urban and rural schools near Dar es Salaam, Tanzania; selection criteria concerned dentine lesions in the occlusal surface that showed no evidence of pulpal involvement; pupils needed to have a dentine lesion present in contralateral permanent molars; infected dentine was removed with slow-speed drill and excavators or by hand excavation with use of Caridex.

**Potential confounders reported: **none.

[5] Parallel group trial. A total of 108 children of the ART group (GIC) and 84 children of the amalgam group were examined at evaluation year 6.3 - from Damascus, Syria; mean age 13.8 years; high risk for dentine lesion development (mean DMFT score 5.5); the mean DMFT and DMFS scores of the children in the ART group were 5.5 (SD 3.0) and 8.2 (SD 5.4) respectively; the mean DMFT and DMFS scores of the children in the amalgam group were 6.0 (SD 1/4 3.3) and 9.4 (SD 6.4); there was no statistically significant difference in caries scores between the children of the two groups (P > 0.05); the mean plaque score for the children in the ART and amalgam group were 1.3 (SD 0.58) and 1.2 (SD 0.52), respectively (see also [3]).

**Potential confounders reported: **Poor oral hygiene; high past caries experience.

[6] Partial split-mouth trial. 45 girls 6-8 years old from a private school (boarding and regular school) in Beirut, Lebanon; from a low socio-economic background with their first and second primary molars requiring new Class I or Class II restorations; specific criteria included vital teeth with normal appearance and morphology, and teeth with or without adjacent teeth; the children routinely (before and during study) received information and instructions to improve their oral hygiene, and had two dental examinations per year; criteria for exclusion from the study: patients having behavioural problems, patients with general health problems, patients with poor oral hygiene, molars requiring pulpotomy or pulpectomy; caries removal with drill;

**Potential confounders reported: **none.

[7] Split-mouth trial. see [4]

[8] Split-mouth trial. 60 Chinese children with mean age 7.4 (SD 1.24) years; 27 boys, 33 girls in Bejing; caries removal for GIC (ART) restorations by hand excavation or drill.

**Potential confounders reported: **none.

[9] Split-mouth trial. 18 caries-active patients, aged 13-16 years; from the regular clientele visiting one of the dental clinics of the Public Dental Health Service in Kronoberg, Sweden; with proximal primary, early carious lesions on contralateral posterior teeth needed restorative treatment; lesion extending into dentin; have progressed into deeper zone since preceding information; caries removal by drill.

**Potential confounders reported: **none.

[10] Partial split-mouth trial. 666 children, from 3 to 13 years of age within the Danish Public Dental Health Service in the municipalities of Vaerløse and Hillerød, Denmark. The caries experience among children and adolescents in the two municipalities is below the national average; caries removal by drill.

**Potential confounders reported: **Low past caries experience

Of the primary datasets, 9 reflected results for restorations in primary teeth, with 3 datasets for single-surface restorations (DS 03,10,11); 2 datasets for multiple-surface restorations (DS 02,04) and 4 datasets (DS 01,07,08,14) for primary teeth in which single- and multiple-surface restorations were combined. Four datasets showed results in permanent teeth: 3 datasets for single surface restorations (DS 05,06,09) and one dataset for multiple surface restorations (DS 12).

The four datasets that showed secondary outcome results were related to caries progression (DS 13,15,16) and regression (DS 17) in approximal tooth surfaces adjacent to restoration surfaces of neighbouring teeth [26,29].

The computed results of each dataset are shown in Table 4.

**Table 4:** Results of individual datasets

Article	DS	RR	95% CI	p-value
Welbury et al., 1991 [20]	01	0.64	0.27 - 1.51	0.31
Östlund et al., 1992 [21]	02	0.73	0.03 - 16.47	0.84
Taifour et al., 2002 [22]	03	0.57	0.24 - 1.36	0.21
	04	0.35	0.11 - 1.10	0.07
Mandari et al., 2003 [23]	05	0.18	0.05 - 0.59	0.005*
Frencken et al., 2007 [24]	06	0.52	0.25 - 1.08	0.08
Daou et al., 2009 [25]	07	4.36	0.51 - 37.09	0.18
	08	0.91	0.21 - 4.04	0.90
Mandari et al., 2001 [27]	09	0.56	0.25 - 1.24	0.15
Yu et al., 2004 [28]	10	Not estimable		
	11	Not estimable		
Svanberg, 1992 [29]	12	0.14	0.01 - 2.53	0.18
	13	0.33	0.12 - 0.91	0.03*
Qvist et al., 1997 [26]	14	0.59	0.28 - 1.25	0.17
	15	0.48	0.32 - 0.71	0.0003*
	16	0.57	0.38 - 0.87	0.0009*
	17	0.91	0.73 - 1.13	0.38

DS = Dataset number; RR = Relative risk; CI = Confidence interval; Not estimable = data from both treatment groups are essentially the same: p = 1.00.

* Statistically significant difference, in favour of GIC.

#### Computed results for restorations in permanent teeth

**Figure 2:**
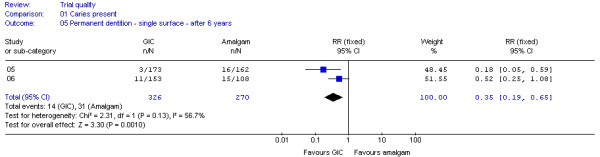
**Forrest plot of meta-analysis results concerning caries on margins of single-surface restorations in permanent teeth after 6 years**. Study or sub-category = Dataset number; GIC = Glass-ionomer cement; RR = Relative Risk; CI = Confidence Interval; n = number of teeth with caries on restoration margins; N = Total number of evaluated teeth.

Of the four datasets, two (DS 05, 06) were considered as fulfilling the criteria for clinical homogeneity [23,24]. Additional analysis established a low statistical heterogeneity (I^2 ^= 56.7%, p = 0.13). For that reason the decision was made to pool both datasets, using a fixed-effects model. The meta-analysis results shown in Figure 2 suggest that margins of single-surface GIC restorations in permanent teeth had a 65% lower chance of developing carious lesions on restoration margins after 6 years than did similar teeth restored with amalgam (RR 0.35; 95% CI 0.19 - 0.65; p = 0.001). No difference was found between single-surface restorations after 1 year (DS 09: RR 0.56; 95% CI 0.25 - 1.24, p = 0.15) [27].

One dataset for multiple-surface restoration (DS 12) was identified. The results of this dataset indicate no difference between the two types of restoration after 2 years (RR 0.14; 95% CI 0.01 - 2.53; p = 0.18) [29].

#### Computed results for restorations in primary teeth

**Figure 3:**
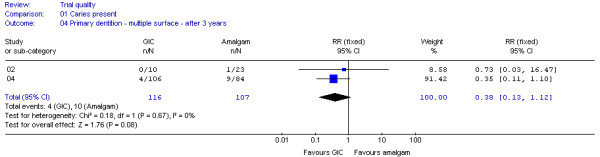
**Forrest plot of meta-analysis results concerning caries on margins of multiple-surface restorations in primary teeth after 3 years**. Study or sub-category = Dataset number; GIC = Glass-ionomer cement; RR = Relative Risk; CI = Confidence Interval; n = number of teeth with caries on restoration margins; N = Total number of evaluated teeth.

Of the nine datasets, two (DS 02,04) were found to fulfil the criteria for clinical homogeneity [21,22]. Additional analysis established a low statistical heterogeneity (I^2 ^= 0%, p = 0.67). For that reason the decision was made to pool both datasets, using a fixed effect model. The meta-analysis results shown in Figure 3 indicate no difference between the types of multiple-surface restorations, with regard to the chance of developing carious lesions on margins after 3 years (RR 0.38; 95% CI 0.13 - 1.12; p = 0.08). All the other datasets also showed no difference between GIC and amalgam in this regard (Table 4).

**Figure 4:**
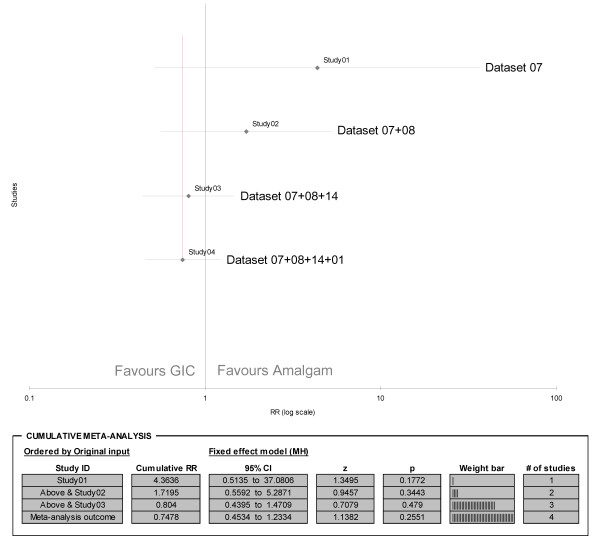
**Forrest plot of cumulative meta-analysis results concerning caries on restoration margins in primary teeth (single- and multiple surface restorations combined)**. RR = Relative Risk; MH = Mantel-Haenszel weight.

In order to investigate whether a possible trend may be assumed in the comparisons of GIC and amalgam, the chronological results from 4 datasets concerning restorations (single- and multiple surface combined) after 1 year (DS 07), 2 years (DS 08), 3 years (DS 14) and 5 years (DS 01) were included in a cumulative meta-analysis [20,25,26]. The datasets were considered homogenous in all aspects except in their follow-up periods. In addition, a lack of statistical heterogeneity was established (I^2 ^= 5.94%; p = 0.36). The cumulative Relative Risk indicates no statistical significant difference between the two materials after 5 years (RR 0.75; 95% CI 0.45 - 1.23; p = 0.26). However, a shift was observed in the cumulative Relative Risk over time, with a continued reduction of the 95% confidence intervals in favour of GIC (Figure 4).

#### Computed results of secondary outcomes for restorations

Three datasets (DS 13,15,16) reported on caries progression, and one dataset (DS 17) reported on caries regression on approximal surfaces of primary or permanent teeth adjacent to each filling material. The results indicate a statistically significant lower caries progression in surfaces adjacent to GIC and showed no difference between the two materials in caries regression after 3 years (Table 4).

### Quality assessment of trial results

#### Selection-, Detection-/Performance bias risk

The results of the quality assessment regarding selection- and detection/performance bias are shown in Table 5. None of the accepted trials reported sufficient details of any randomisation process that had indeed given each patient the same chance to be allocated to either the GIC or the amalgam group and to ensure that direct observation and prediction of the allocation sequences was successfully prevented. Only two trials [22,24] had reported baseline data collected before randomisation and reported for both treatment groups, statistically compared this data between groups and found the difference statistically not significant (p > 0.05). No accepted trial reported on successful blinding/masking of patients, operators and trial evaluators.

**Table 5:** Results of quality assessment of accepted trials

Article	DS	Selection bias		Detection/Performance bias	Attrition bias		Trial outcome
			
		Randomisation	Baseline data	Blinding/Masking	Loss-to-follow up	Run-in phase	
Welbury et al., 1991 [20]	01	0	0	0	A	A	A
Östlund et al., 1992 [21]	02	0	0	0	B	A	A
Taifour et al., 2002 [22]	03	0	A	0	A	A	A
	04	0	A	0	A	A	A
Mandari et al., 2003 [23]	05	0	0	0	B	A	A
Frencken et al., 2007 [24]	06	0	A	0	A	A	A
Daou et al., 2009 [25]	07	0	0	0	A	A	A
	08	0	0	0	A	A	A
Mandari et al., 2001 [27]	09	0	0	0	B	A	A
Yu et al., 2004 [28]	10	0	0	0	A	A	A
	11	0	0	0	A	A	A
Svanberg, 1992 [29]	12	0	0	0	A	A	A
	13	0	0	0	A	A	0
Qvist et al., 1997 [26]	14	0	0	0	B	A	A
	15	0	0	0	A	A	0
	16	0	0	0	A	A	0
	17	0	0	0	A	A	0

DS = Dataset number.

#### Attrition bias risk

All datasets were computed under the assumption that either (i) all restored teeth lost to follow-up developed caries on margins or (ii) no restored teeth lost to follow-up developed caries on margins. The results did not change the conclusions for the majority of datasets. However, a possible risk of attrition bias was identified in the results of three datasets (DS 02, 05, 14) extracted from three trials [21,23,26]. The results of sensitivity analysis showed:

(i) For dataset 02 [21]: If it were assumed that all restored teeth lost to follow-up had developed caries on restoration margins, the result would be significantly in favour of amalgam (RR 5.00; 95% CI 1.65 - 15.15; p = 0.004);

(ii) For dataset 05 [23]: If it were assumed that all restored teeth lost to follow-up had developed caries on restoration margins, the result would show no significant difference between the two treatment groups (RR 0.81; 95% 0.59 - 1.11; p = 0.18);

(iii) For dataset 14 [26]: If it were assumed that all restored teeth lost to follow-up had no caries progression on tooth surfaces adjacent to either material, the result would not show a significant difference between the two treatment groups (RR 0.68; 95% CI 0.32 - 1.44; p = 0.32).

In line with the potential influence of attrition bias on datasets 02 and 05, the meta-analysis results (Figure 2 and 3) would change for single-surface restorations in permanent, and multi-surface restorations in primary teeth to RR 0.90; 95% CI 0.83 - 0.98; p = 0.01 and RR 1.02; 95% CI 0.95 - 1.09; p = 0.67 respectively, if it were assumed that all restored teeth lost to follow-up had developed caries on margins

In addition to the risk of bias due to loss-to-follow-up, no trial indicated that a run-in phase was implemented before randomisation (Table 5).

#### Publication bias risk

**Figure 5:**
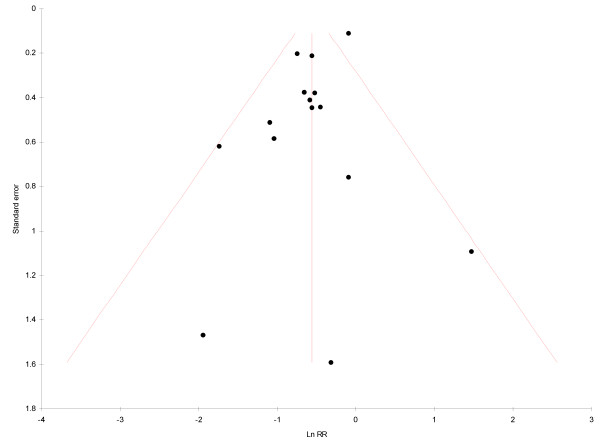
**Funnel plot of dataset results (test for publication bias)**. RR = Relative Risk.

Publication bias was investigated, using one funnel plot (Figure 5). The funnel plot covering data for caries progression showed an even distribution that did not suggest publication bias. Egger's linear regression method for the same datasets showed an intercept of 0.96 (95% CI -2.03 - 0.11; p = 0.07). The regression result was not statistically significant.

## Discussion

The aim of this quantitative systematic review was to update the existing evidence related to the review question about whether, on margins of restored tooth cavities in the same dentition and of same cavity class, GIC-restored cavities show less recurrent carious lesions than cavities restored with amalgam.

The new systematic literature search found 12 more trials [25-36] that could be included for review. This was possible because of the publication of one new trial since the original search cut-off date; a more thorough hand-search and reference check of the literature, and broader inclusion criteria. Of the 12 new included trials, 7 were excluded [30-36], as they did not comply with the stated exclusion criteria. One trial [19] that was accepted in the originally published systematic review [7] was now excluded, as it did not report on loss-to-follow-up restorations per treatment group, which that made it impossible to discern the total number of evaluations (N) at the end of the follow-up period. Therefore, it was also not possible to use sensitivity analysis to assess the potential risk of attrition bias for this trial.

In comparison to the original published systematic review [7], this update presents an improvement in the output of its systematic literature search. However, other aspects in the methodology of this review update might still have contributed to limitations in its results: (i) not all relevant publications were listed in the selected databases; (ii) The chosen search terms may not have been broad enough; (iii) not all relevant publications could be found through hand-search and reference check. Furthermore, we decided to limit inclusion of trials found through hand-search and reference check to trials published from 1990, only. Thus, some relevant studies published before 1990 would not have been identified. However, a plausible basis for the hypothesis that GIC may contain caries preventive characteristics was only developed in the mid-Nineties [4]. Trials that investigated the characteristics of GIC before that period may therefore not contain observations concerning caries on restoration margins as primary outcomes. Such assumption is confirmed by the fact that the majority of trials excluded from this review due to lack of reported outcome measure related to caries were published before 1995 [31,34,35].

### Selection-, Detection-/Performance bias risk

All of the accepted trials appear to be limited by risk of selection- and detection/performance bias. Bias or systematic error may affect studies, causing either an over- or an under-estimation of the treatment effect of an investigated clinical procedure. Overestimation has been observed to be the most common [37]. Kjaergard et al. reported a treatment effect overestimation of 48% caused by lack of random sequence allocation [38] and Egger et al. reported a treatment effect overestimation of 54% and 53% due respectively to lack of allocation concealment and lack of evaluator blinding [39].

It has been emphasized that selection bias can be successfully prevented only if the allocation sequence remains truly random and free from potential interference throughout the trial [9,10]. For this reason it is important that trials include an effective process for concealing the random allocation sequence and that such process has indeed prevented direct observation and prediction of the random sequence allocation throughout each trial [9,10]. Quality assessment in terms of the internal validity of trials should, therefore, be a measure of the result of random sequence allocation and allocation concealment, and not only of its reported attempt. All trials accepted in this systematic review failed to report not only on evidence of successful sequence allocation and allocation concealment results but also on necessary details about how sequence allocation and allocation concealment was attempted (Table 5). None of the trials therefore provide any guarantee that each patient had an equal chance to be allocated to either treatment group and thus their allocation may have favoured the outcome of one type of treatment above the other. One measure for testing whether random sequence allocation has been successful is testing whether covariates differ between treatment groups at baseline [9]. Only two articles, reporting different datasets from the same trial, had included such a test and reported on its outcome [22,24]. The statistically non-significant results (p > 0.05) suggest a successful random allocation. However, doubt remains regarding potential bias risk, as other non- balanced covariates may exist that were not tested for and/or not reported.

From the onset, successful blinding or masking appeared not to have been possible in all trials, owing to the obvious differences between GIC and amalgam in their clinical appearance. For that reason the allocation to either treatment group was visible to patients, operators and evaluators. However, the difficulties of successful blinding still carry the danger of detection-/performance bias, which thus may have affected the results of the trials. Potential knowledge of superiority claims prior to the trial may have led patients to change their oral hygiene habits, operators to place restorations more carefully or evaluators to apply evaluation criteria more subjectively. This in turn may have favoured the outcome of one type of treatment above the other.

### Attrition bias risk

Sensitivity analysis may be used to establish whether missing data could have affected the trial outcomes, by assuming that the numbers of restoration lost to evaluation were either failures or successes [40]. Comparison of the resulting analysis results with reported trial outcomes indicates whether different conclusion should be drawn. Sensitivity analysis was conducted for all datasets. The analysis results differed from reported outcomes of three datasets (DS 02,05,14) extracted from three trials [21,23,26]. According to the analysis results, multiple-surface restorations placed with GIC (DS 02) would have a 5-times higher chance than amalgam of developing caries on restoration margins after 3 years in primary teeth [21] and single-surface restorations placed with GIC (DS 05) would have only a 10% (instead of 65%) lesser chance of developing caries on margins than amalgam after 6 years in permanent teeth [23]. However, neither would affect the overall conclusions drawn from the meta-analyses (Figures 2 and 3): that single-surface GIC restorations have a lesser likelihood of developing caries on margins in permanent teeth after 6 years and that no difference can be found between the two materials regarding multiple-surface restorations after 3 years in primary teeth.

The outcome of dataset 14 would be affected by attrition bias, in that no significant differences between GIC and amalgam would be found if all patients lost to follow-up were assumed to show no caries progression in the adjacent teeth [26]. However, a statistically significant difference would remain in favour of GIC (p = 0.002) if all lost patients were assumed to show caries progression. It has been observed that trials with patient exclusion showed more beneficial test treatment effects than analyses based on randomisation of all, or most, patients [41]. This may be due to the possibility that patients drop out or discontinue treatment if the treatment proves unsuccessful. As no proof is available about how many patients who were unavailable for evaluation really did or did not have caries progression, the more conservative assumption would be that most of them had progressed caries. Against such a background, a remaining statistical significant difference between the treatment groups, in favour of GIC, seems more likely.

A run-in phase is considered to be a stage during a trial where all patients receive, for example, the test treatment and only those patients that respond well to the treatment are later used for random allocation in either the control or the test group [10]. Such practice would effectively exclude patients from the randomisation process and potentially favour, for example, the test group above the control. No such run-in phase was indicated in any of the accepted trials (Table 5).

### Publication bias risk

Publication bias was investigated by generating a funnel plot (Figure 5). Publication bias is present when the results of published research differ from those of all the research that has been done [42]. Funnel plots are scatter graphs showing the size of studies on the Y-axis (large studies on top, small studies at the bottom) and the effect size, observed in these studies, on the X-axis. The effect sizes of larger studies have the tendency to cluster near the mean. Small studies have effect sizes that are dispersed across a wider range. Results of both types of studies, plotted on a scatter graph, give the shape of an inverted, in absence of publication bias symmetrical, funnel [43]. Publication bias affects a funnel plot in the form of a concentration of studies to only one side (asymmetry). Such asymmetry is created when particular smaller studies are published only when they show a larger than average effect. However, if the number of studies (n) is less than 10, any asymmetry may be due to chance and not to publication bias [44]. For that reason the decision was made to plot results of the 17 extracted datasets instead of those of published articles. Despite this departure from the common use of funnel plots, the use of datasets (instead of published studies) will also indicate potential publication bias when only datasets that show a larger than average effect are published and other datasets are not. In this update, the funnel plot concerning dichotomous RCT data on caries progression showed a symmetrical spread of dataset results (Figure 5). As the visual judgement of funnel plots is subjective, intercepts were calculated (95% CI), using Eggers regression [14]. The calculated intercept confirmed a non-significant result. Eggers regression is used to quantify bias captured by funnel plots [43]. However, its reported power is considered to be low unless in the presence of severe bias or a high number (n) of studies/datasets (n >10) [43,44]. Therefore, the results of the calculated intercept concerning datasets for caries progression (n = 17) may be ascribed to lack of severe publication bias.

### Data extraction and analysis results

The extended scope of this update did not change the overall results of the meta-analysis originally published [7]. However, it has to be noted that these results are limited by risk of selection- and detection-/performance bias. As the true extent of such bias impact remains unknown within the reviewed trials, the results need to be regarded with caution.

In eight out of the ten accepted trials that used split-mouth or partial split-mouth design (Table 3) a cross-over effect caused by fluoride, released from the GIC restoration, may have reduced the caries susceptibility of tooth margins surrounding the amalgam restorations and thus may have confounded the observed results towards a more equivocal outcome.

It also has to be noted that none of the accepted studies reported on fluoride exposure of subjects. It can be assumed that subjects may have been exposed to external fluoride sources and that this may have increased the caries resistance of teeth restored with amalgam, thus confounding the caries-preventive effect of GIC as suggested by Hara et al. [45]. In this context, it is worth pointing out that the only trial reporting on a statistically significant primary outcome in favour of GIC was conducted in a developing country in Africa [23], where the opportunities of exposure to external fluoride sources may be few. The hypothesis that GIC restorations may in general be less susceptible to recurrent caries is supported by the secondary trial outcomes (DS 13,15,16) [26,29].

A significantly lower chance of caries development on GIC restoration margins was reported in the permanent dentition only [23]. In the primary dentition, the results for multiple-surface restorations after 3 years (Figure 3) suggest that neither of the materials is superior. The reasons for this remain unclear. It can be assumed that factors like the larger restoration surface, as well as the greater difficulties involved in placing restorations in children than in adults, may outweigh any caries-preventive properties of GIC in comparison to amalgam. However, under ideal trial conditions free of bias and confounder influence, GIC restorations may also have a lower chance of developing caries on restoration margins on a long-term basis in primary teeth. This statement should be regarded as a hypothesis requiring testing through high quality randomised control trials. Plausibility for this hypothesis is provided by the results of the included cumulative meta-analysis (Figure 4). Cumulative meta-analysis is used to provide insights into how much the efficacy of treatments, often reported as mean results with 95% confidence intervals, change over time as evidence accumulates [46]. It is the result of conducting a new meta-analysis each time a new set of evidence emerges [47]. A cumulative meta-analysis not only allows the evaluation of any additional contributions made by individual studies to the cumulatively combined results of preceding studies [48] but also allows observation of a trend in evidence direction over time. The forest plot in Figure 4 shows a steady shift in cumulative results, starting after 1 to 5 years, in favour of GIC. The observed shift is caused by a cumulative change in Relative Risk (from 4.36 to 0.75) and the narrowing of the cumulative 95% confidence interval (from 0.51 - 37.08 to 0.45 - 1.23).

### Recommendations for further research

Systematic reviews have been reported as providing the highest form of clinical evidence [49]. However, the internal validity of such evidence can only be as good as the internal validity of the trials reviewed. Although the trials accepted in this update may be considered not much affected by attrition- and publication bias, their risk of selection- and detection/performance bias is high. Thus, further high quality randomised control trials (RCT) are needed in order to verify (or disprove) the currently available results. Such RCTs should adopt a parallel group design and include randomisation and allocation concealment methods that can effectively prevent direct observation and prediction of the allocation sequence. For this purpose the maximum randomisation method has been suggested [9]. Covariates of both treatment groups should be tested as to whether they differ at baseline (after randomization). Recently, the inclusion of the Berger-Exner test has been recommended for authors of trials needing to investigate whether selection bias has been introduced into their studies [9,10]. Where bias risk has been found, such risk may be adjusted statistically [9]. In order to assure that the lack of blinding may not have led to the favouring of one treatment above another, trials should use and report on procedures and tests employed that may have limited, or at least monitored, potential bias risk. Future trials should also base their reporting on the CONSORT statement [50].

## Conclusion

The results of this update of previous systematic evidence confirm the findings of the original published review regarding whether, on margins of restored tooth cavities in the same dentition and of same cavity class, GIC-restored cavities show less recurrent carious lesions than cavities restored with amalgam. Although the findings in this update may be considered less affected by attrition- and publication bias, their risk of selection- and detection-/performance bias is high. Therefore, further high-quality randomised control trials are needed in order to verify the currently available results.

## Competing interests

The authors declare that they have no competing interests.

## Authors' contributions

Both authors contributed equally to the systematic literature search, review, data extraction and the writing of the manuscript. SM conducted the data analysis.
